# Beliefs About Medicines and Adherence to Treatment in Turkish Patients with Inflammatory Bowel Disease

**DOI:** 10.5152/tjg.2022.21355

**Published:** 2022-09-01

**Authors:** Güray Can, Ahmet Yozgat, Ahmet Tezel, Gülbin Ünsal, Ali Rıza Soylu

**Affiliations:** 1 Department of Gastroenterology, Abant İzzet Baysal University Faculty of Medicine, Bolu, Turkey; 2 Department of Gastroenterology, Ufuk University Faculty of Medicine, Ankara, Turkey; 3 Department of Gastroenterology, Trakya University Faculty of Medicine, Edirne, Turkey

**Keywords:** Adherence, Crohn’s disease, inflammatory bowel disease, medication, treatment, ulcerative colitis

## Abstract

**Background:**

Although studies are investigating the perception and beliefs about treatment and adherence to treatment in different societies related to inflammatory bowel disease, there are no studies on this subject in Turkish people with different sociocultural structures. In our study, we aimed to evaluate the beliefs about treatment and its effect on adherence to treatment in the Turkish population with inflammatory bowel disease.

**Methods:**

In the study, the “Medication Adherence Report Scale” and “Beliefs about Medicines Scale” scales were used to evaluate the treatment compliance and perception and beliefs about treatment. Characteristics that could affect treatment compliance were evaluated by statistical analysis.

**Results:**

A total of 253 patients, 167 with ulcerative colitis and 86 with Crohn’s disease, were included in the study. The non-adherence rate to the treatment was found as 41.9% in ulcerative colitis and 24.4% in Crohn’s disease (*P =* .006). Intentional (29.3% in ulcerative colitis and 16.3% in Crohn’s disease [*P* = .031] and unintentional non-adherence to treatment (28.1% in ulcerative colitis, 16.3% in Crohn’s disease [*P* = .037] were significantly higher in ulcerative colitis than in Crohn’s disease. Female gender (odds ratio = 2.59, *P* = .005), low education level (odds ratio = 4.8, *P* = .015), distal involvement in ulcerative colitis (*P* = .014), and thoughts about the disease would last too soon in Crohn’s disease (odds ratio = 4.17, *P* = .049) were risk factors for non-adherence to treatment.

**Conclusion:**

The negative perception of treatment in inflammatory bowel disease affects adherence to the treatment. Considering some social factors that affect adherence to the treatment and taking measures to enhance the adherence to treatment will increase the success of treatment.

## Introduction

Inflammatory bowel disease (IBD) is a group of diseases with chronic inflammation that affects the gastrointestinal system, with remission and exacerbation periods, and the etiology has not yet been clarified very well.^1,2^ Due to the natural course of IBD, it is necessary to continue the treatment for an imprecise period.^3,4^ While mesalamine is highly effective in remission induction, maintenance, and chemoprevention of colorectal cancer in the treatment of ulcerative colitis (UC),^5^ immunomodulatory drugs constitute the basis of the treatment in Crohn’s disease (CD). Non-adherence to treatment significantly increases the risk of relapse and colorectal carcinoma (especially in UC patients), increases healthcare costs,^6^ and even disability in IBD^7^; so continuity of treatment is important. Beliefs of medication, including doubts about the effects of the drugs and their necessity, and concerns about potential side effects are the basis of non-adherence to treatment.^8^

Adherence to treatment can be defined as the rate of cooperation between the physician’s recommendations and the patient’s behavior.^9^ While the effectiveness of treatment increases adherence to treatment in patients, the younger age of the patients, potential side effects, and long duration of treatment have a negative effect on adherence.^10^ Non-adherence to the treatment, which is around 30%-40% in IBD patients,^11^ significantly affects the effectiveness and the prognosis of the treatment.^12^ Many clinical and sociodemographic factors such as the duration of the disease, the form, doses of the drugs used, marital status, and gender are associated with non-adherence to treatment.^13^ Besides, the patient–doctor relationship, psychological stress factors, the doctor’s knowledge about the adherence, and the use of objective approaches affect the adherence to treatment.^10,13^

Non-adherence to treatment is divided into 2 categories: intentional and unintentional. Unintentional non-adherence is the inability of the patient to receive treatment due to reasons such as forgetfulness, limitation in understanding the treatment scheme, and physical and financial inadequacy despite the patient’s desire to receive treatment, whereas intentional non-adherence is not taking the treatment as recommended by his/her own decision due to reasons such as the perception of treatment and personal preferences.^14^ Both non-adherence categories are frequently encountered in IBD.^15,16^ A detailed description of the underlying causes of non-adherence to treatment will enhance the success of interventions that will increase adherence.

On the other hand, depending on the socioeconomic level of the patients, the sociocultural structure of the society, the psychosocial characteristics of the patient, their general positive and negative perceptions, and experiences about drugs also affect their beliefs about the treatment given for IBD. Although there are many studies in different societies, there is no study evaluating the perception of treatment in IBD and its effect on adherence to treatment in Turkish people which has different sociocultural, religious, and belief structures. There are several validated tests that evaluate the adherence to treatment in patients, among which the most commonly used are the Medication Adherence Report Scale (MARS) and Morisky Medication Adherence Scale (MMAS-8)^17^ scales, and the MARS^18^ scale was used in this study. In the present study, we aimed to evaluate the beliefs about treatment and its effect on adherence to the treatment in a Turkish population with IBD.

## Materials and Methods

### Patient Selection and Ethical Issues

Patients older than 18 years of age who were followed up with the diagnosis of CD and UC in the gastroenterology outpatient clinics of Trakya and Abant İzzet Baysal University Faculty of Medicine were included in the study during 3 months period after the ethical approval of the study. Patients with indeterminate colitis, mental problems, who could not understand the scales, and who did not want to participate in the study were excluded from the study. The study was approved by the Trakya University Faculty of Medicine Non-Interventional Clinical Research Ethics Committee (decision number: 25/16, Date: 19.12.2012). Informed consent was obtained from all patients participating in the study.

### Validation of Scales

Since the “MARS” and “Beliefs about Medicines Questionnaire” (BMQ)^14^ used in the study were not validated in Turkish, the scales were first translated into Turkish and then back-translated into English by 2 different native speakers of English. The obtained translations were sent to the survey owner at his request and were approved by him.

### Study Design

Medication Adherence Report Scale was used to measure adherence, and the BMQ scale was used to evaluate the perception of treatment in patients who were routinely followed up and received any treatment for IBD (mesalamine, sulphasalazine, corticosteroid, azathioprine, infliximab, or adalimumab).^14,18^ In addition to these, they were asked to fill in a structured form containing clinical, demographic, and treatment-related parameters that may affect adherence to treatment. They were asked whether they talked to others about the disease, how long the disease would last, and how much they knew about the disease. Crohn’s disease Activity Index in CD and Mayo scoring in UC were used to evaluate disease activity at the time of admission. All of the scales were administered to outpatients just before the outpatient clinical examination by a nurse who had no function in treating the patients.

### Assessment of Adherence to Medication

In the MARS scale, 5 different behavioral patterns such as “I forget to take the drugs” and “I change the dosage of the drugs” were evaluated, and the scores were collected on the 5-point Likert-type scale and a total score between 5 and 25 was obtained. The score of the question “I forget to take the drugs” was evaluated as the intentional non-adherence, and the scores of the other 4 questions were evaluated as the unintentional non-adherence. The presence of either intentional or unintentional non-adherence was evaluated as non-adherence to treatment, and the absence of both as adherence to treatment.

### Assessment of Belief of Medication

The BMQ scale consisting of 2 parts was applied to the patients to evaluate their perceptions about the treatment given specifically for IBD and to all medical treatments in general. In the first part, the perception of necessity and concern of the patients were evaluated on a 5-point Likert-type scale and the average perception was determined by calculating the midpoint. Values above the midpoint indicate strong perception in the relevant scale, and lower values indicate a weaker perception. A necessity-concerns differential (NCD) was calculated by subtracting the individuals’ concerns scores from the individuals’ necessity scores, leading to a range from −20 to 20. In the second part of the BMQ, the perceptions of the patients about the nature of the drugs, in general, were evaluated as beneficial, harmful, or overused.

### Statistical Analysis

Mann–Whitney *U*-test was used to evaluate the relationship between clinical and demographic variables with each behavioral pattern in the MARS scale and each BMQ score. One-way analysis of variance was used to compare the concern and necessity scores of the drugs used with the MARS scores. Tukey test was used as a post hoc test. The correlation between BMQ scores and risk factors was evaluated using the Spearman test. Multivariate regression analysis was performed to test the relationship between drug adherence and risk factors independently. Chi-square test was used in the comparison of categorical data; Fisher’s exact test was applied if appropriate. The CI was calculated as 95%. *P* = .05 was accepted as significant. Data were analyzed using the Statistical Package for Social Sciences version 20.0 software (IBM Corp.; Armonk, NY, USA).

## Results

A total of 280 patients were invited to the study, and a total of 253 IBD patients, 167 with UC and 86 with CD, were included in the study. There was no significant difference between UC and CD patients in terms of the mean age (44.9 ± 14.2 vs 44.6 ± 14.5 *P =* .88) and gender distribution. In the internal consistency analysis of the scales, Cronbach’s α values were 0.75 for UC, 0.70 for CD in BMQ, 0.76 for UC, and 0.75 for CD in MARS.

Primary school graduates were in the majority in both diseases (50.3% in UC and 52.3% in CD). There was no significant difference between the 2 diseases in terms of origin, place of residence, occupation distribution, and education level. Smoking was reported in 10.8% of UC and 25.6% of CD patients (*P* = .009). The mean body mass index in CD was significantly higher than in UC; it was 25.4 ± 4.1 and 23.1 ± 4.3 (*P* = .001). Whereas the most common involvement in UC patients was left type with 44.3%, it was ileocolonic (58.1%) in CD. During the survey, the proportion of patients with active disease was higher in UC, and the rate of those who had an IBD-related operation was higher in CD (*P* = .025 and *P* = .001, respectively). The number of outpatient and inpatient visits for IBD last year and the time since the last outpatient visit and last colonoscopy were similar between the 2 diseases. The results regarding the drugs used by the patients, their usage patterns, and other clinical features are shown in Table 1.

When the MARS scores were compared, unintentional non-adherence was 28.1% in UC, 16.3% in CD (*P* = .037), and intentional non-adherence was 29.3% in UC, and 16.3% in CD (*P* = .031). Low treatment adherence (non-adherence) was found as 41.9% in UC and 24.4% in CD (*P* = .006) (Table 2).

The rate of those who thought the necessity of treatment in both diseases was over 80% and the rate of those who thought that drug treatment was beneficial was around 70%. The rate of those who thought that drug treatment was harmful (around 20%) or overused medication (around 24%) was lower. Besides, the rate of those who had concerns about IBD treatment was found to be 40%. No significant difference was found between the 2 diseases in terms of necessity, concern, overused medication, and harmful scores (Table 3).

**Figure 1.**
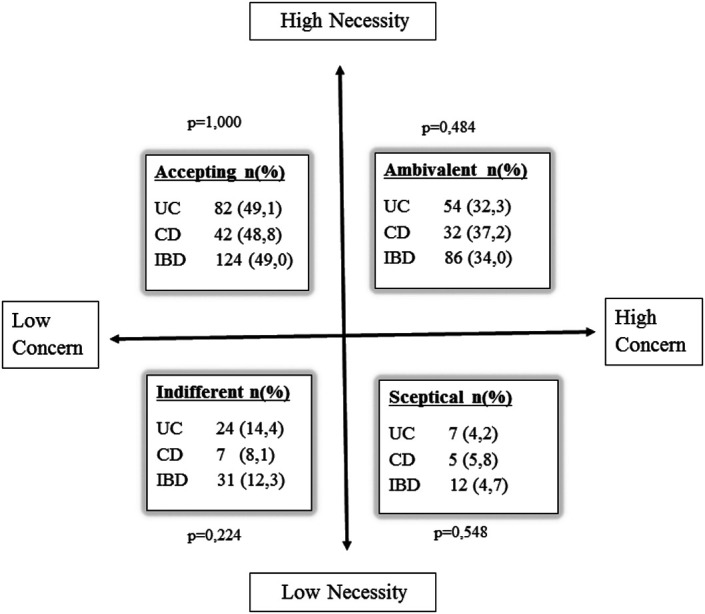
Attitudinal analysis of beliefs about maintenance treatment in inflammatory bowel disease. UC, ulcerative colitis; CD, Crohn’s disease; IBD, inflammatory bowel disease.

**Figure 2.**
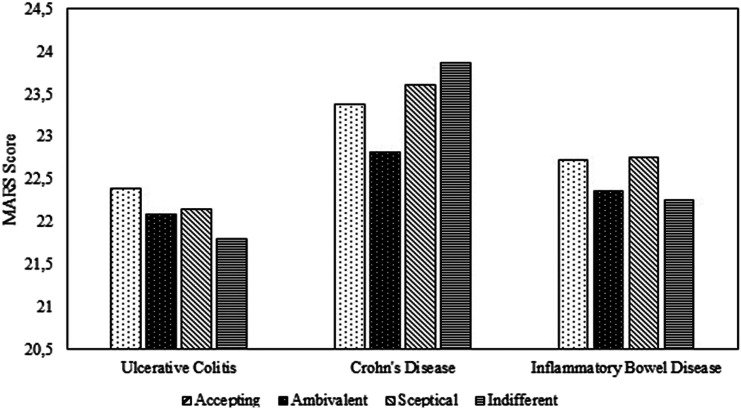
Adherence to treatment across attitudinal patterns (ulcerative colitis, *P* = .85; Crohn’s disease, *P* = .64; inflammatory bowel disease, *P* = .76). MARS, Medication Adherence Report Scale.

Approximately 50% of the patients in both UC and CD had an “accepting” attitudinal pattern (*P* = 1.00), while the “ambivalent” pattern was 32.3% in UC and 37.2% in CD (*P* = .48). The rates of “skeptical” and “indifferent” groups were low in both diseases. Specific attitudinal pattern distribution was similar in both diseases (*P* = .45) (Figure 1). When the MARS scores of 4 different attitudinal patterns were compared, the scores in CD were higher than the scores in UC, but a significant difference was observed only between the “indifferent” groups (UC, 21.8 ± 3.4 and CD, 23.8 ± 1.1, *P* = .01) (Figure 2).

In both diseases, necessity and concern were not effective on adherence to the treatment. Harm perception was higher in patients with low adherence in both UC and CD than those with high adherence (2.68 ± 0.80, 2.22 ± 0.77, *P* = .0001 for UC and 2.86 ± 0.99, 2.27 ± 0.77, *P* = .006 for CD). The overused perception was higher in patients with low adherence in CD than those with high adherence (2.93 ± 0.68 vs 2.5 ± 0.57, *P* = .03).

There was a significant negative correlation between perception of harm in UC (*r* = −0.25, *P* = .003) and the perception of concern, harm, and overused medication in CD and adherence to treatment (*r* = −0.22, *P* = .04; *r* = −0.29, *P* = .007; and *r* = −0.25, *P* = .02, respectively). A positive correlation was found between the necessity and beneficial perception in both UC and CD (*r* = 0.53, *P* = .0001 and *r* = 0.59, *P* = .0001; respectively) (Table 4).

Multivariate logistic regression analysis was performed to evaluate the effect of demographic, clinical, and social variables in predicting adherence to the treatment. Being female (odds ratio (OR) = 2.59, *P* = .005), being illiterate (OR = 4.8, *P* = .015), having distal-type involvement (*P* = .014), not knowing the disease at all (OR = 2.61, *P* = .042) in UC, dealing with friends, (OR = 4.54, *P* = .038), and thinking that the disease will last too soon (OR = 4.17, *P* = .049) in CD was determined as a risk factor for low adherence to the treatment (Table 5).

In both diseases, the perception of necessity was higher than the perception of concern for all types of drugs used. Although the perception of the necessity and concern for corticosteroids in UC and the necessity of azathioprine in CD was higher, the concern of azathioprine in CD was lower than other types of drugs; there was no significant difference between the drug types in both UC and CD (UC; necessity *P* = .35, concern *P* = .22; CD, necessity *P* = .24, concern *P* = .34). When the NCD scores of the drug types used in both diseases were compared between the low and high adherence to treatment groups, no significant difference was found for any drug type. Medication Adherence Report Scale scores were higher in patients who used corticosteroids in UC, biologics, and then corticosteroids in CD, than in those who used other drugs. But, there was no significant difference among drugs in both diseases (MARS scores; *P* = .58 for UC, *P* = .48 for CD).

## Discussion

This study provides qualitative information about beliefs on treatment and its effects on adherence to treatment in Turkish patients with IBD which has a different religious, ethnic, and sociocultural society structure. Low adherence to treatment was 41.9% in our UC patients and 24.4% in our CD patients. The rate of intentional and unintentional non-adherence among the non-adherence patients was approximately equal in UC and CD (29.3 vs 28.1; 24.9 vs 24.1, respectively). Both types of non-adherence to treatment (*P* = .031 vs *P* = .037) and total non-adherence to treatment (*P* = .006) were significantly higher in UC patients. The fact that IBD patients are generally young, their quality of life is close to normal in the inactive disease stages, the risk of reactivation cannot be predicted, and the need for long-term treatment are the reasons that cause low adherence to treatment in these patients.^19,20^ Despite the wide spectrum of non-adherence to treatment rates in IBD patients in many studies, the average rate is between 30% and 50%,^21^ while in a Korean study this rate was 22.7%,^20^ in a recent study in China, non-adherence to oral treatments was found to be 37%.^22^ These differences in adherence to treatment between societies can be explained by education level and other social dynamics.

The mean age of our patients is 44 years in both diseases, and it is higher than a few studies in recent years (e.g., 38.3 in Kim et al’s^20^; 33 in Hu et al’s^22^ studies), which is a variable that can affect the beliefs about medication and adherence to treatment. In the study of Kim et al^20^ adherence to treatment was found to be less in younger patients, while our patients’ older age and lower education levels may lead to lower treatment compliance.

In the present study, non-adherence to treatment was significantly higher in UC patients than in CD patients. The differences in the perception of the necessity for treatment in terms of drugs may be important among the factors that affect this situation because the perception of the necessity for corticosteroids is higher in UC and azathioprine is higher in CD than other drugs. The use of 5-aminosalicylic acid (ASA) derivatives (the need to take many drugs during the day) in the maintenance treatment of UC and the fact that the patients are completely normal during remission periods may also contribute to the difference in non-adherence levels between the 2 diseases.

In our study, rates of intentional and unintentional non-adherence to treatment were found to be close to each other in both diseases. While unintentional non-compliance was found to be higher in the study conducted by Kim et al^20^ rates similar to our results were found in the study conducted by Cerveny et al.^15^ It is important to teach treatment regimens, the risks of complications, and the consequences of non-adherence to treatment to reduce rates of unintentional non-adherence.^20^ This is possible with a better patient–physician relationship.

When the attitudinal patterns were evaluated in our patients, it was found that accepting and ambivalent attitudes were higher than skeptical and indifferent attitudes in both diseases. However, when the MARS scores of 4 different behavioral patterns were compared, no significant difference was found between the groups in both diseases. Among the diseases, the rate of non-adherence was found to be significantly higher in the indifferent group only in CD compared to UC. In the study of Horne et al^8^ lower rates of adherence to treatment were found in patients with skeptical, ambivalent, and indifferent attitudinal patterns, whereas a study by Kim et al^20^ found no difference between the groups similar to our study.

When the beliefs about the treatment of patients with low adherence to treatment were evaluated, it was found that harm perception in both diseases and overused medication perception in only CD negatively affected treatment compliance. These results are consistent with the finding that negative beliefs cause non-adherence to treatment.^23^ This suggests that some patients may have the perception that “I am well for years, but they are still giving much treatment to me.” This perception can only be overcome by increasing the level of education about the disease.

As expected in our study, the perception of harmful in UC patients and harmful, concern, and overused medication perception in CD patients were negatively correlated with adherence to treatment. Also, adherence to treatment was positively correlated with patients who have the necessity and beneficial perception in both diseases.

In many studies, the predictors affecting low adherence to treatment were evaluated, and it was determined that young age, small bowel lesions, side effects of drugs, unemployment, indifferent and skeptical attitude, psychological distress, physician–patient discordance, being in remission, and intensive work are important.^11,13,20,22^ In the study of Keil et al^24^ adherence to treatment was found to be only associated with education level in UC patients using 5-ASA and it was stated that patient preferences should be taken into account in order to improve the level of adherence.^24^ In our study, it was determined that being a woman, being illiterate, having distal-type involvement, and not knowing the disease in UC patients, on the other hand, dealing with friends and thinking that the disease will last very soon in CD patients were the predictors of non-adherence to treatment. While it is expected that dealing with friends increases adherence to treatment, the opposite result in our study suggests that society has a factor effect that attenuates the perception of the disease on the patient. Besides, this non-adherence to treatment may be due to the stigmatization seen in many infectious diseases. To overcome these reasons, it is necessary to carry out educational activities about disease not only on patients but also on at least in the patient’s close circle.

When the limitations of our study should be addressed, first, it is important that our work was conducted in a certain region, included a limited number of patients not representing the whole country. The educational level, cultural, and religious structure of the region where the study was conducted may be effective in adherence to treatment but was not evaluated in our study. The fact that quantitative methods were not used in the evaluation of adherence to treatment is another limitation of the study. Further prospective studies evaluating a larger number of patients are needed.

In conclusion, this is the first study that evaluates the beliefs about treatment and its effects on adherence to treatment in the Turkish population. The negative perception of treatment in IBD affects adherence to treatment. Considering some social, cultural, religious, and environmental factors that affect adherence to treatment and taking measures to enhance adherence to treatment will increase the success of treatment.

## Figures and Tables

**Table 1.** Sociodemographic and Clinical Characteristics of the Inflammatory Bowel Disease Patients

	Ulcerative Colitis	Crohn’s Disease	*P*
Number (n)	167	86	-
Age (year, mean ± SD)	44.9 ± 14.2	44.6 ± 14.5	.880
Gender (female), n (%)	74 (44.3)	39 (45.3)	.870
Location (ulcerative colitis), n (%)			-
Proctitis	21 (12.6)	-	
Left-sided colitis	74 (44.3)	-	
Pancolitis	72 (43.1)	-
Location (Crohn’s disease) n (%)			-
Ileal	-	25 (29.1)	
Colonic	-	11 (12.8)	
Ileocolonic	-	50 (58.1)
Behavior (Crohn’s disease), n (%)			-
Non-stricturing–non-penetrating	-	56 (65.1)	
Stricturing	-	21 (24.4)	
Penetrating	-	9 (10.5)
IBD family history, n (%)	27 (16.2)	6 (7.0)	.110
Age of diagnosis (year, mean ± SD)	38.2 ± 13.9	39.9 ± 14.6	.360
Disease activity (active patients), n (%)	51 (30.5)	15 (17.4)	**.025**
Time since diagnosis of IBD (mo, mean ± SD)	79.2 ± 89.5	54.9 ± 44.5	**.004**
Total time at remission (mo, mean ± SD)	12.6 ± 22.8	17.2±27.3	.158
Total time on the treatment (mo, mean ± SD)	60.0 ± 71.6	44.2 ± 39.8	**.025**
IBD operation (at least 1 time), n (%)	8 (4.8)	30 (34.9)	**.0001**
Number of IBD drugs, n (%)			**.0001**
One	51 (30.5)	51 (59.3)	
Two	89 (53.3)	24 (27.9)	
Three and more	15 (9.0)	4 (4.7)
Total daily drug doses (mean ± SD)	6.7 ± 3.8	6.8 ± 4.1	.970
Side effect of IBD drugs (at least 1 time), n (%)	59 (35.3)	37 (43.0)	.232
Daily medication frequency, n (%)			**.0001**
Once	13 (8.2)	19 (22.9)	
Twice	31 (19.5)	18 (21.7)	
Thrice	54 (34.0)	39 (47.0)	
Four times	61 (38.4)	7 (8.4)
High awareness about disease, n (%)	110 (65.9)	49 (57.0)	.166
Chronicity belief about disease, n (%)	135 (80.8)	69 (80.2)	.908

IBD, inflammatory bowel disease; Mo, month; SD, standard deviation.

**Table 2.** Comparison of the Medication Adherence Between the Patients of Ulcerative Colitis and Crohn’s Disease

	Ulcerative Colitis	Crohn’s Disease	IBD	*P*
MARS total (mean ± SD)	22.20 ± 3.07	23.22 ± 2.41	22.55 ± 2.90	**.004**
Unintentional non-adherence, n (%)	47 (28.1)	14 (16.3)	61 (24.1)	**.037**
Intentional non-adherence, n (%)	49 (29.3)	14 (16.3)	63 (24.9)	**.031**
Non-adherence level, n (%)				**.006**
Low adherence	70 (41.9)	21 (24.4)	91 (36.0)	
High adherence	97 (58.1)	65 (75.6)	162 (64.0)

MARS, Medication Adherence Report Scale; IBD, inflammatory bowel disease; SD, standard deviation.

**Table 3.** Comparison of the Beliefs About Inflammatory Bowel Disease Medication and Medicines in General Between Ulcerative Colitis and Crohn’s Disease

BMQ	BMQ Subscales	Ulcerative Colitis	Crohn’s Disease	IBD	*P*
Specific	Necessity, n (%)	136 (81.4)	74 (86.0)	210 (83.0)	.302
	Mean ± SD	3.87 ± 0.97	3.99 ± 0.78	3.91 ± 0.91
	Concern, n (%)	61 (36.5)	37 (43.0)	98 (38.7)	.253
Mean ± SD	2.77 ± 0.81	2.90 ± 0.86	2.81 ± 0.83
General	Harmful, n (%)	33 (19.8)	17 (19.8)	50 (19.8)	.992
	Mean ± SD	2.41 ± 0.81	2.41 ± 0.87	2.41 ± 0.83
	Overused, n (%)	41 (24.6)	20 (23.3)	61 (24.1)	.895
	Mean ± SD	2.62 ± 0.76	2.60 ± 0.80	2.61 ± 0.77
	Beneficial, n (%)	116 (69.5)	62 (72.1)	178 (70.4)	.522
Mean ± SD	3.51 ± 0.91	3.58 ± 0.87	3.53 ± 0.90

BMQ, Beliefs About Medication Questionnaire; IBD, inflammatory bowel disease.

**Table 4.** Pearson Correlations Between Adherence to Treatment, Beliefs About Inflammatory Bowel Disease Medications and Beliefs About Medicines in General

	BMQ Subscales	Ulcerative Colitis		Crohn’s Disease		IBD	
*r*	*P*	*r*	*P*	*r*	*P*
MARS total	Necessity	0.072	.36	0.060	.58	0.079	.21
	Concern	−0.041	.59	−0.221	**.041**	−0.080	.20
	Beneficial	−0.048	.53	−0.016	.89	−0.032	.62
	Harmful	−0.225	**.003**	−0.289	**.007**	−0.238	**.0001**
Overused	−0.098	.21	−0.249	**.021**	−0.141	**.025**
Necessity	Beneficial	0.535	**.0001**	0.594	**.0001**	0.550	**.0001**
	Harmful	−0.024	.76	−0.285	**.008**	−0.102	.10
Overused	0.060	.44	−0.080	.46	0.017	.78
Concern	Beneficial	−0.056	.47	−0.146	.18	0.084	.18
	Harmful	0.306	**.0001**	0.327	**.002**	0.313	**.0001**
Overused	0.241	**.002**	0.245	**.023**	0.241	**.0001**

MARS, Medication Adherence Report Scale; BMQ, Beliefs about Medication questionnaire; IBD, inflammatory bowel disease.

**Table 5.** Associated Clinical and Demographic Factors in Predicting Non-adherence to Treatment

Group	Variables	*P*	Exp (*B*)	95% (CI) Min-Max
UC	Gender (F)	.005	2.59	1.39-4.83
	Illiterate	.015	4.8	1.81-28.2
	Distal-type involvement	.005	4.13	1.53-11
Not knowing the disease	.042	2.61	1.03-6.61
CD	Dealing with friends	.038	4.54	1.08-18.52
Thinking that the disease will last too soon	.049	4.17	1.01-16.67

UC, ulcerative colitis; CD, Crohn’s disease; F, female; Multivariate logistic regression analysis.
